# Highly Luminescent Transparent Cs_2_Ag_*x*_Na_1–*x*_Bi_*y*_In_1–*y*_Cl_6_ Perovskite Films Produced by Single-Source Vacuum Deposition

**DOI:** 10.1021/acsmaterialslett.3c00034

**Published:** 2023-01-19

**Authors:** Oleksandr Stroyuk, Oleksandra Raievska, Paz Sebastia-Luna, Bas A. H. Huisman, Christian Kupfer, Anastasia Barabash, Jens Hauch, Henk J. Bolink, Christoph J. Brabec

**Affiliations:** † Forschungszentrum Jülich GmbH, Helmholtz-Institut Erlangen Nürnberg für Erneuerbare Energien (HI ERN), 91058 Erlangen, Germany; ‡ Insituto de Ciencia Molecular, Universidad de Valencia, Catedrático J. Beltrán 2, 46980 Paterna, Spain; § Friedrich-Alexander-Universität Erlangen-Nürnberg, Materials for Electronics and Energy Technology (i-MEET), Martensstrasse 7, 91058 Erlangen, Germany

## Abstract

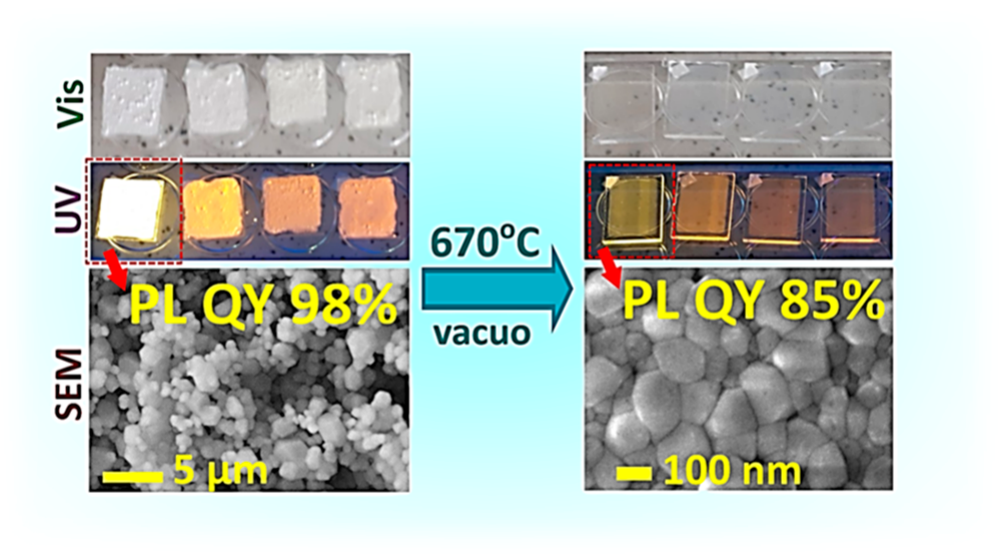

Thermal deposition of halide perovskites as a universal and scalable route to transparent thin films becomes highly challenging in the case of lead-free double perovskites, requiring the evaporation dynamics of multiple metal halide sources to be balanced or a single-phase precursor preliminary synthesized to achieve a reliable control over the composition and the phase of the final films. In the present Letter, the feasibility of the single-source vacuum deposition of microcrystalline Cs_2_Ag_*x*_Na_1–*x*_Bi_*y*_In_1–*y*_Cl_6_ double perovskites into corresponding transparent nanocrystalline films while preserving the bulk spectral and structural properties is shown. The perovskite films produced from the most emissive powders with *x* = 0.40 and *y* = 0.01 revealed a photoluminescence quantum yield of 85%, highlighting thermal evaporation as a promising approach to functional perovskite-based optical materials.

Lead-free halide perovskite semiconductor compounds feature an inspiring combination of structural variability with a plethora of promising functional properties, in particular, those related to the absorption and emission of light.^1−7^ Some double-halide perovskites, A_2_^I^M^I^M^III^X_6_ (A – alkali metal, M^I^ + M^III^ – isovalent substitution of a Pb^II^ + Pb^II^ pair in conventional lead perovskites, X = halide), are considered highly promising phosphors (X = Cl)^1−3,6−8^ and absorbers (X = Br, I) in LEDs and photovoltaic devices, respectively.^2,5^ The double-halide perovskites based on Bi^III^, Sb^III^, In^III^, etc. reveal a unique mechanism of radiative recombination via the formation of a self-trapped-exciton (STE) state, resulting in broadband photoluminescence (PL) emitted in the visible and NIR spectral ranges.^1,2,6,9^ The STE emission of many double-halide perovskites shows an unprecedented compositional and structural variability of spectral parameters as well as high PL quantum yields (QYs), in some cases approaching 100%.^10−17^

Recently we have reported on a green synthesis route to microcrystalline lead-free double perovskites with a general formula Cs_2_Ag_*x*_Na_1–*x*_Bi_*y*_In_1–*y*_Cl_6_ (CANBIC) emitting broadband self-trapped-exciton PL in the visible spectral range centered at ca. 600 nm.^16,17^ The perovskite synthesized with *x* = 0.3–0.4 and *y* = 0.01–0.02 showed a champion room-temperature PL QY of 98 ± 2% as well as a prominent environmental stability.^17^ The combination of a strong absorption at λ < 400 nm, a highly intense PL in the range of 500–800 nm with almost no overlap between absorption and the PL band, as well as environmental, thermal, and photochemical stability, and synthetic availability of this compound make it a promising material for optical applications, including luminescent down-shifting solar concentrators and components of displays.^1−3^ At the same time, microcrystalline CANBICs are characterized by intense light scattering, limiting their direct applicability as phosphors.

Transparent CANBIC-based materials can potentially be produced by many different methods, including those minimizing light scattering on the grain boundaries (sintering/pressing of microcrystals, the introduction of a binder with a comparable refractive index, etc.) or reducing the grain size to the nanometer range,^8,18,19^ for example, by introducing appropriate ligands during the synthesis or subjecting CANBIC powders to a mechanochemical treatment or thermal recrystallization.^20^

The thermal evaporation/recrystallization of complex halide perovskites can potentially be performed in different regimes, including sequential evaporation and coevaporation from several sources as well as single-source deposition.^20^ In the case of CANBIC, where five metal chloride sources are required, the multisource deposition is expected to be highly challenging due to differences in the evaporation rates and the possibility of the formation of multiple phases with simpler composition.

Considering the scalability, reproducibility, and reliable compositional control achieved by us recently in the synthesis of microcrystalline CANBICs,^16,17^ the single-source thermal evaporation using these powders as stable and available precursors would be a much more promising route to transparent perovskite layers as compared to the multisource approaches. At the same time, the thermal deposition of luminescent CANBIC perovskites as transparent films and the evolution of their spectral properties upon the conversion from the powders to films have not been reported so far.

In the present Letter, we confirm the feasibility of single-source vacuum deposition (SSVD) of microcrystalline CANBICs in the form of stable transparent nanocrystalline films on glass substrates. We show that, despite the complexity of CANBIC composition and rather high sublimation temperatures, the transfer from light-scattering microcrystalline powders to transparent nanocrystalline films can be performed for any Bi-to-In ratio while preserving the original stoichiometry and phase uniformity of the perovskites as well as their light-emissive capacity. The presented results highlight the SSVD approach as a potential gateway opening numerous optical applications for microcrystalline lead-free halide perovskite phosphors.

The lead-free double CANBIC perovskites emit intense broadband self-trapped-exciton PL in the visible spectral range peaking at ca. 600 nm, the PL intensity and spectral parameters depending on both Ag (*x*) and Bi (*y*) fractions, which can be varied independently.^16,17^ The CANBICs crystallize in a cubic *Fm3m*-type lattice typical for In–Bi-based double perovskites, forming a series of ideal solid solutions with the lattice parameter gradually increasing from 10.523 Å for *y* = 0.01 to 10.823 Å for *y* = 1.00 (Table 1).^17^ The highest PL QY of 98 ± 2% was detected for a CANBIC compound with nominal *x* = 0.4 and *y* = 0.01–0.02 showing an almost single-exponential PL decay profile with an average PL lifetime of ca. 2 μs.^17^ As the bismuth content in CANBIC is increased, the absorption band edge and PL band shift to longer wavelengths, while the PL efficiency and PL lifetime are drastically reduced, to 5% and ∼130 ns, respectively (Table 1), for Cs_2_Ag_0.4_Na_0.6_BiCl_6_ perovskite (*y* = 1.00).^17^

**Table 1:** Lattice Parameter *L*, PL QY, LO Phonon Frequency *v*_LO_, and FWHM *w*_LO_ for CANBIC Powders and Thin Films Produced at Different Bi Fractions *y*^a^

	*L* (Å)		PL QY (%)		*v*_LO_ (cm^–1^)		*w*_LO_ (cm^–1^)	
*y*	powder	film	powder	film	powder	film	powder	film
0.01	10.523	10.522	98	85	299.0	299.2	14	17
0.50	10.671	10.683	14	11	288.9	289.1	20	25
0.90	10.788	10.784	7	4	283.6	283.6	20	26
1.00	10.823	10.816	5	3	282.7	282.8	20	24

a Measurement accuracy is 0.005 Å (*L*), 2% (PL QY), and 0.1 and 1 cm^–1^ (*v*_LO_ and *w*_LO_).

Based on our previous results,^16,17^ a series of microcrystalline CANBIC powders with a fixed Ag content at *x* = 0.40 and a varying Bi content *y* from 0.01 to 1.00 were selected to test the feasibility of thermal-evaporation-induced deposition of transparent CANBIC films on glass substrates. We used a method similar to that published previously (more details provided in Experimental Methods below).^21,22^

Figure 1a shows a collection of photographs of the microcrystalline CANBIC powders and corresponding thermally deposited thin films with four different *y*, taken at ambient conditions and under UV illumination. The films are totally transparent in transmitted light but appear to be semitransparent under UV illumination due to rather a strong emission of yellow-to-brown PL depending on the bismuth content. The emission colors are similar to those observed from the powders. The films revealed prolonged stability retaining constant spectral properties (absorption and PL spectra, data not shown) when stored for at least two months in ambient conditions without encapsulation.

**Figure 1:**
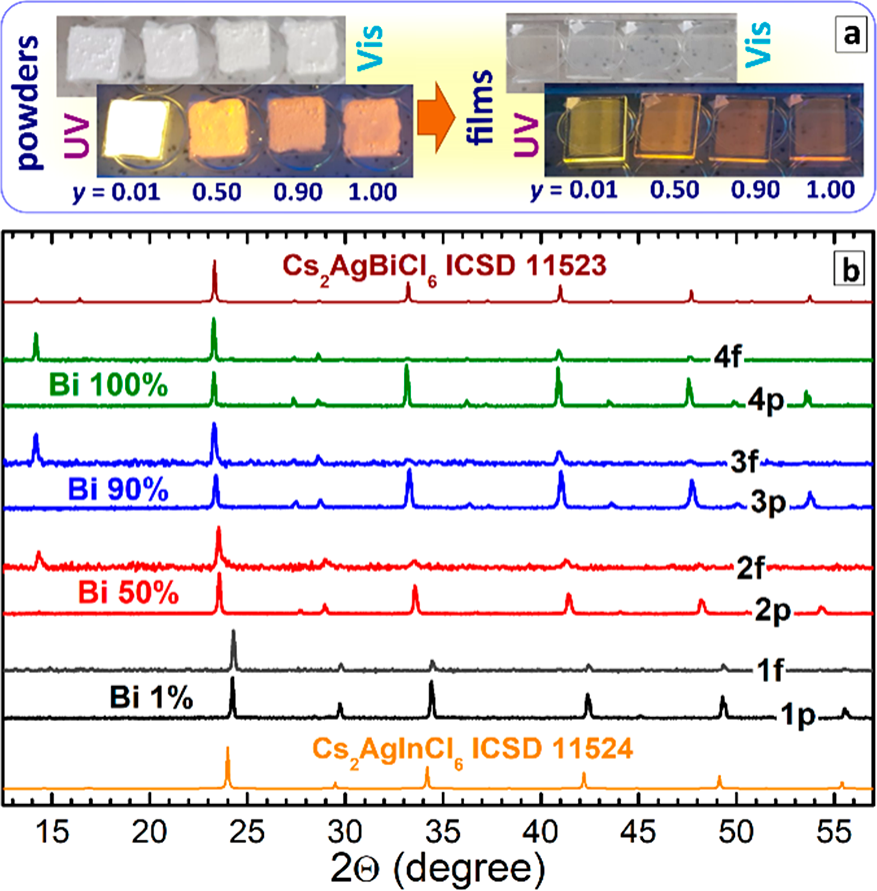
(a) Photographs of original CANBIC powders and thermally evaporated CANBIC films taken under ambient and UV illumination (360–370 nm). (b) XRD patterns of CANBIC powders (curves 1p–4p) and thin films (1f–4f) produced at *y* = 0.01 (1), 0.50 (2), 0.90 (3), and 1.00 (4); XRD patterns are shifted along *Y*-axis for better visibility.

The original CANBIC powders and corresponding films showed almost identical XRD patterns (Figure 1b) and lattice parameters (Table 1), revealing no additional phases or other signs of CANBIC decomposition during the deposition. Considering the compositional complexity of CANBIC perovskites as well as the number of possible secondary phases, this result can be regarded as an extraordinarily positive indication of the perspectives of the thermal evaporation for the transfer of highly luminescent CANBIC phosphors from strongly light-scattering powders to transparent and uniform thin films.

The CANBIC films grown from powders with 50 and 100% Bi (Figure 1b, curves 2f, 3f) showed a change in the intensity of the diffraction peaks compared to those from the original powders. In particular, the films revealed a much higher intensity of (111) and (220) diffractions at 2θ of ca. 15 and 24°, not typical for the corresponding bulk phases.

These differences can be regarded as an indication of the oriented growth of CANBIC NCs in the thin films, in accordance with the microscopic observations discussed below.

Similar strong redistributions of diffraction peaks were also reported for lead halide perovskite crystals cut/grown along different crystallographic axes^23−25^ as well as for lead-free double-perovskite nanosheets.^26^

Raman spectroscopy is a powerful tool for the identification of the composition and phase transformations of CANBIC perovskites,^16,17^ which can provide evidence for the presence of the perovskite phase and allows the Bi-to-In ratio to be estimated from the frequency of the longitudinal optical (LO) phonon peak. A comparison of Raman spectra of bulk and thermally evaporated CANBIC samples (Figure 2a) showed the powders and the films to be identical in terms of the phase and composition, showing the same frequencies of LO phonon peaks (Figure 2b, *v*_LO_ in Table 1). As compared to the powder samples, the corresponding films showed somewhat higher fwhm’s of the LO peak (*w*_LO_ in Table 1), most probably indicative of residual compressive stress in the films due to the lateral restriction of the crystal growth during the film formation, as discussed further. We note that the present results highlight Raman spectroscopy as a promising control tool for the express identification of the phase and composition of thermally deposited lead-free double-perovskite films.

**Figure 2:**
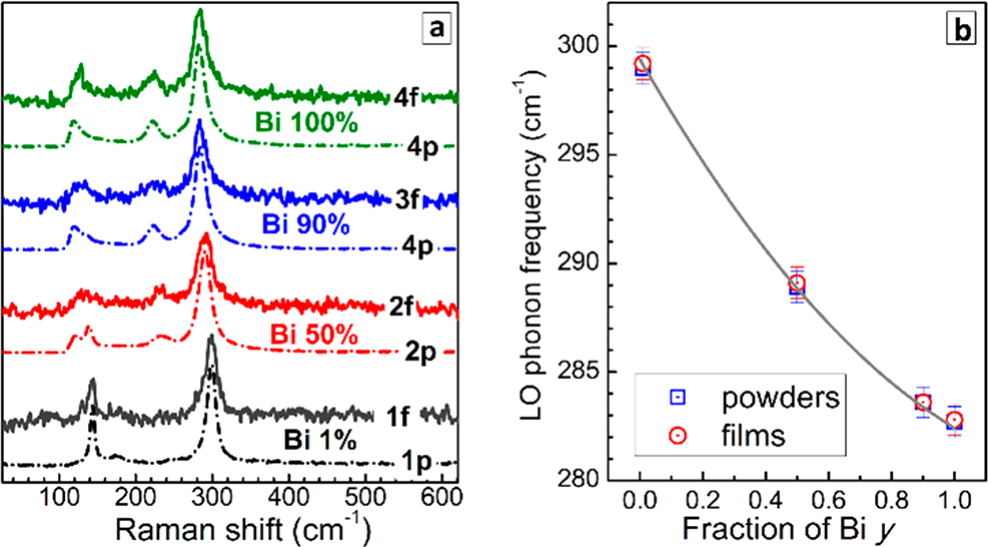
(a) Raman spectra of CANBIC powders (dashed lines 1p–4p) and films (solid lines 1f–4f) with *y* = 0.01 (1), 0.50 (2), 0.90 (3), and 1.00 (4). Spectra are shifted along *Y*-axis for better visibility. (b) LO phonon frequency of CANBIC powders (squares) and films (circles) as a function of Bi fraction *y*. The gray solid line represents a calibration curve for the determination of CANBIC composition from Raman spectra reported in ref (17).

The CANBIC powders used for the SSVD are composed of randomly aggregated microcrystals with a grain size in the range of 1–3 μm for *y* = 0.01 (Figure 3a) and decreasing for Bi-richer compositions (Figure 3c,e). A scanning electron microscopy (SEM) inspection of the surfaces of the thermally deposited films showed them to have considerably different morphology, being composed of densely packed grains with a size of 100–200 nm (Figure 3b,d,f). Considering that the thickness of the films was found to vary in the range of 250–300 nm, the films can be assumed to be formed by a single layer of closely packed single CANBIC nanocrystals (NCs), elongated along the direction normal to the substrate surface due to lateral growth restriction during the SSVD.^20^

**Figure 3:**
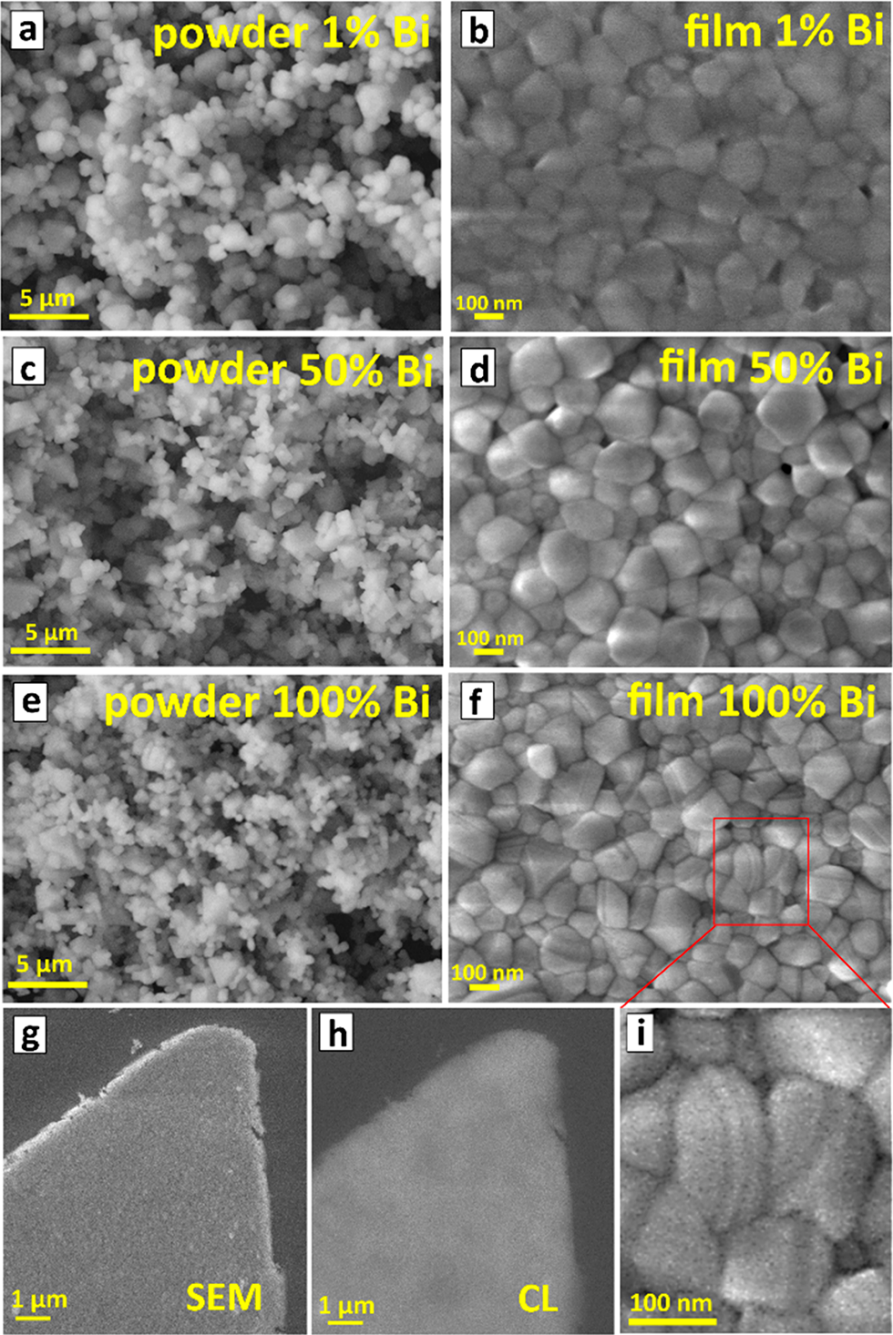
(a–f) SEM images of CANBIC powders (a,c,e) and thin films (b,d,f) produced at *y* = 0.01 (a,b), 0.50 (c,d), and 1.00 (e,f). (g,h) Microscopic images of CANBIC film with *y* = 0.01 registered using SEM contrast (g) and CL contrast (h). (i) shows an enlarged section of SEM image (f) indicated by a red frame.

Similar to earlier reported microcrystalline CANBICs,^16,17^ the thermally deposited films revealed strong cathodoluminescence (CL) when probed by an electron beam during SEM measurements. By using CL as a contrast-forming signal, the morphology of the films can be visualized in the same way, as by using secondary electron contrast (Figure 3h,g), showing a reasonable uniformity of the thermally deposited films in terms of the composition and thickness.

The CANBIC NCs in the films with 1–50% Bi (Figure 3b,d) showed no specific trends in grain morphology, the grains having a random polyhedral shape dictated partially by the lateral growth restriction. At the same time, the films deposited from powders with 100% Bi (Figure 3f) revealed a visually discernible layered grain structure, the crystals resembling “sandwiches” composed of flat nanoplates (see a magnified fragment in Figure 3i). As no considerable broadening of the XRD reflections was observed for these films, the nanoplates can be argued to be single-crystalline and not composed of thinner sheets. The preferred growth of CANBC (*y* = 1.00) crystals in the form of nanoplates can account for the much more pronounced (111) and (220) reflections observed for the films with *y* = 0.05 and 1.00 as compared to the corresponding powders. We note that a more detailed insight into the effects of the oriented growth of CANBIC NCs in the thermally deposited films goes beyond the scope of the present Letter and will be presented in more detail elsewhere.

Analysis of absorption and PL spectra of the thermally deposited CANBIC films clearly showed that the spectral characteristics of CANBIC perovskites are mostly transferred from the micro- to nanocrystalline state without major changes. Both powders and films showed identical positions of the absorption band edges, indicative of the same bandgap for the different CANBIC compositions (Figure 4a,b). At the same time, we note that the powder sample with *y* = 1.00 shows an absorption peak at ca. 330 nm, which is absent in the absorption spectrum of the corresponding film but still can be observed in the PL excitation spectrum of the same film. At the moment, we have no explanation for these observations, and additional research is currently being performed with different film thicknesses and Bi contents to get deeper insights into the relationship between the absorption properties of bulk and corresponding nanocrystalline CANBIC perovskites.

**Figure 4:**
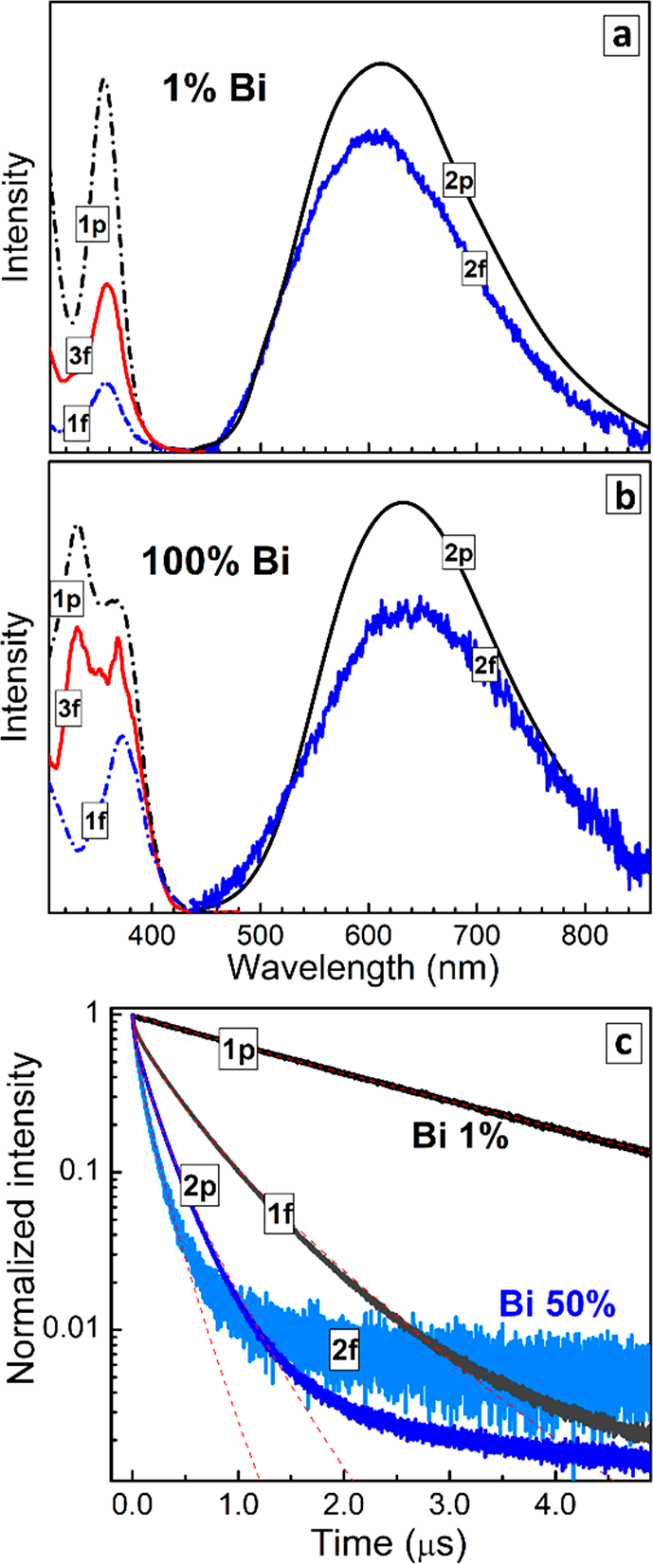
(a,b) Absorption (dashed lines 1) and PL (solid lines 2) spectra of CANBIC powders (1p, 2p) and films (1f, 2f); PL excitation (red lines 3f) spectra of thin films are included; *y* = 0.01 (a) and 1.00 (b). (c) Normalized kinetic curves of PL decay of CANBIC powders (solid lines 1p, 2p) and films (solid lines 1f, 2f) with *y* = 0.01 (1) and 0.50 (2). Red dashed lines show the fitting of PL decay curves with a stretched-exponential function.

The shape and position of the PL band remain mostly unaffected as well. Minor differences in the PL band shape can be accounted for by a low integral PL intensity of the films due to either a very low absorbance (as in the case of 1% Bi) or a low PL QY (as in the case of 100% Bi). PL excitation spectra of the films coincide with the corresponding absorption spectra even for the highest Bi content, indicating that no decomposition of the perovskite into a mixture of luminescent and nonluminescent products occurred during the SSVD.

The PL QY of the film produced from the most luminescent CANBIC sample (*x* = 0.40, *y* = 0.01), measured at room temperature by excitation with a 365 nm light source, was found to be 85% (Table 1), indicating only a minor loss of the PL efficiency during the perovskite transfer from microcrystalline powder to a nanocrystalline thin film. To the best of our knowledge, the present PL QY is the highest one reported for a semitransparent nanocrystalline film of a lead-free double perovskite.

The films with a higher Bi content expectedly showed lower PL QYs, but in all cases, only a minor loss in the emission efficiency is observed for the films as compared to the original microcrystalline powders. This fact additionally highlights the SSVD as a promising tool for manufacturing highly emissive transparent perovskite layers.

In contrast to the stationary PL behavior, when both powders and films of CANBIC revealed a strikingly similar behavior, the PL decay kinetics of micro- and nanocrystalline samples was found to be rather different (Figure 4, Table 2). While the most emissive 1% Bi CANBIC sample showed an almost single-exponential PL decay profile (curve 1 in Figure 4c) with an average PL lifetime of 2.35 μs, the corresponding nanocrystalline film exhibited a strong reduction in the PL lifetime down to ca. 450 ns and a strong deviation from the single-exponential character of the decay trace (curve 2 in Figure 4c), indicative of additional nonradiative recombination pathways, inactive in the bulk material.

**Table 2:** Average PL Lifetime (τ), Rate Constants of Radiative (*k*_r_) and Nonradiative (*k*_nr_) Recombination, and Heterogeneity Factor (*h*) for CANBIC Powders and Thin Films Produced at Different Bi Fractions *y*^a^

	τ (ns)		*k*_r_ × 10^5^ (s^–1^)		*k*_nr_ × 10^5^ (s^–1^)		*h*	
*y*	powder	film	powder	film	powder	film	powder	film
0.01	2350	445	4.2	19.1	0.1	3.4	1.05	1.86
0.50	330	140	4.2	7.9	26.1	63.6	1.37	1.56
0.90	160	90	4.4	4.4	58.1	106.7	1.35	1.43
1.00	135	85	3.7	3.5	70.4	114.1	1.24	1.33

a Measurement accuracy is 5 ns (τ), 0.1 × 10^5^ s^–1^ (*k*_r_, *k*_nr_), and 0.01 (*h*).

Fitting of the PL decay trace with a stretched-exponent function, similar to our previous reports,^16,17^ allowed also the heterogeneity parameter *h* to be evaluated, which reflects the perfection of lattice and energy distribution of the self-trapped-exciton state of the perovskite.^9^ The *h* parameter is very close to 1 for the bulk 1% Bi CANBIC indicating a high uniformity of the perovskite lattice with a very narrow distribution of possible STE energies. The heterogeneity parameter of the corresponding film increases almost twice, to 1.9 (Table 2), clearly showing the effects of lattice distortion in the nanocrystalline CANBIC. Many factors can be responsible for this effect, resulting in the reduction of the PL lifetime and PL QY, the most obvious ones being internal stress, affecting the energy and dynamics of the self-trapped-excitonic state as well as generation of bulk and surface defects in the thermally deposited CANBIC films. Such factors affecting PL QY and recombination dynamics were already discussed for lead halide perovskites^20,27^ but still have to be recognized and properly addressed in the case of thermally evaporated lead-free analogs.

Both trends of the PL lifetime reduction and decay shape change are general and observed for all tested compositions, see Table 2 and exemplary curves for 50% Bi CANBIC in Figure 4c (curves 3, 4). An increase in the Bi content in CANBICs enhances the nonradiative recombination pathways,^17^ resulting in a lower PL lifetime and PL QY in the bulk materials. At that, the differences in the PL decay dynamics between bulk CANBICs and corresponding films gradually become smaller, almost leveling off for the 100% Bi CANBIC (Table 2).

The rate constants of radiative recombination (*k*_r_) and nonradiative recombination (*k*_nr_) can be evaluated from the average PL lifetime and PL QY using the well-known expressions *k*_r_ = (PL QY)/τ and τ^–1^ = *k*_r_ + *k*_nr_. The values of the recombination rate constant calculated for all tested powders and films are presented in Table 2. In the case of CANBIC powders, the rate of radiative recombination was found to be almost independent of the Bi content, while *k*_nr_ increased by ca. 3 orders of magnitude with the Bi fraction elevated from 1% to 100% (Table 2).

The nanocrystalline CANBIC films with 1% and 50% Bi showed a considerable increase in the radiative recombination rate as compared to the powder precursors, while at higher Bi contents (90% and 100%), *k*_r_ remains almost unaffected by the powder-to-film transition (Table 2). At the same time, the rate of nonradiative recombination was found to be strongly enhanced in all tested CANBIC films as compared to the corresponding powders.

In the case of the most luminescent 1%Bi sample, the radiative recombination in the film is ca. 5 times faster than in the corresponding powder. At the same time, the rate of nonradiative recombination is increased by a factor of 34 as the powder is converted into the film (Table 2). The simultaneous growth of both *k*_r_ and *k*_nr_ can account for the fact that 1%Bi CANBIC film retains a high PL QY of 85% at a strongly reduced average PL lifetime.

The enhancement of nonradiative processes in CANBIC films stems most probably from a higher density of bulk and surface defects as compared to the original powder. The simultaneous acceleration of the radiative recombination can be associated with the presence of strain in closely packed CANBIC nanocrystals, resulting in an additional lattice distortion and facilitating the formation of self-trapped-excitonic states.^9^

In summary, we show that vacuum sublimation is a suitable method to obtain transparent nanocrystalline thin films from microcrystalline powders of CANBIC double perovskites. The films have a thickness of ca. 200 nm and are virtually identical in composition and phase to the starting powder materials. The transparent CANBIC films were found to retain the same spectral parameters of absorption and PL bands and structural characteristics (composition, phase uniformity, lattice parameter) as the original powders with a wide range of Bi-to-In ratios varying from 0.01 to 1.00. The CANBIC films are composed of nanocrystals with a lateral size on the order of 100 nm, in contrast to the original bulk CANBIC samples characterized by a grain size of 1–3 μm.

The CANBIC films produced from the most luminescent CANBIC powders (*y* = 0.01, PLQY of 98%) exhibited a PL QY of 85%, which is to the best of our knowledge the highest value reported for a transparent nanocrystalline film of a lead-free double perovskite so far. The feasibility of the transfer of CANBIC double perovskites from strongly scattering microcrystalline powders to transparent and uniform nanocrystalline films while preserving the composition, phase uniformity, and high PL efficiency highlights that vacuum thermal evaporation is a promising approach to functional lead-free-perovskite-based optical materials. This approach benefits from both the green character of the synthesis of starting CANBIC powders and the single-source scheme of the thermal evaporation allowing the material/energy input to be minimized and high control over the composition and structure of the final perovskite films to be retained.

## Experimental Methods

### Samples

Microcrystalline CANBIC perovskites were synthesized according to the earlier reported protocol^16,17^ via rapid mixing of two precursor solutions, containing a varied amount of Bi^III^ + In^III^ (first precursor) and Ag^I^ + Na^I^ + Cs^I^ (second precursor) in a mixed 2-propanol/water solvent at ambient conditions. The as-prepared precipitates were purified by multiple centrifugations in 2-propanol, dried in the air, and kept in ambient conditions.

### Thermal Evaporation/Deposition

Deposition of thin films was performed by single-source vacuum deposition inside a vacuum chamber at a pressure of ca. 8 × 10^–6^ mbar.^21^ The distance from the crucible to the substrate holder was approximately 15 cm. In a typical procedure, 0.10–0.15 g of perovskite powders was placed into an Al_2_O_3_ crucible located in a bell jar-type vacuum chamber. Glass-based substrates were added in a substrate holder to which a shadow mask can be fitted. After pumping down the pressure in the bell jar, the crucible was heated rapidly to a temperature of 670 °C, which was found to be the evaporation temperature of the whole CANBIC series. By setting the crucible temperature directly at the evaporation temperature of the material, we aimed to prevent decomposition into unwanted side products and the evaporation of the compound as a whole. The sublimation rate was monitored by a quartz crystal microbalance. The process finished when there remained no powder in the crucible, followed by an abrupt decrease in the sensor reading. Then, the source temperature was cooled down, and the chamber was vented to ambient pressure. It must be noted that venting of the chamber and handling of the samples take place under atmospheric conditions. Films of an average thickness of 250–300 nm were obtained. No further annealing of the films was performed.

### Structural Characterization

The X-ray diffraction (XRD) patterns of the powders and films were collected using a Panalytical X’pert powder diffractometer with filtered Cu*K*_α_ radiation (λ = 1.54178 Å) and an X’Celerator solid-state stripe detector in the Bragg–Brentano geometry in an angle range of 2θ = 5–100° with a step rate of 0.05° per min. The samples of microcrystalline CANBICs for XRD were prepared by drop-casting of suspensions on glass and dried at ambient conditions. The XRD patterns were subjected to the Rietveld refinement procedure using MAUD software. The thickness of the thermally deposited films was determined by a mechanical profilometer Ambios XP200. SEM and CL imaging were performed using a JEOL JSM-7610F Schottky field emission scanning electron microscope operating at an accelerating voltage of 15–20 kV and equipped with a Deben Centaurus detector (for CL). The samples for SEM/CL were prepared by drop-casting a suspension in 2-propanol onto polished adhesive carbon tape attached to a single-crystalline silicon plate and dried at ambient conditions. CL images were registered at the lowest sensitivity of the detector by integrating the emission in the entire spectral range.

### Spectral Characterization

Reflectance spectra of CANBIC powders and absorption spectra of CANBIC films were recorded using a BlackComet spectrometer (StellarNet) and a 75 W xenon lamp (Thorlabs) as an excitation source. Absorption spectra of powders were calculated by dividing the reflectance spectra of the reference and the sample (Spectrolon, StellarNet) and subtracting a baseline. PL spectra were registered with a BlackComet spectrometer in the range of 190–1000 nm using a UV LED (360–370 nm, Thorlabs) as an excitation source. PL excitation spectra were taken using a Tecan fluorescence spectrometer equipped with a Xe lamp and a monochromator. Photographs of luminescent CANBIC perovskites were taken at ambient conditions under illumination with a UV lamp (350–370 nm). PL QY of the transparent films was determined using a UV–vis–NIR Absolute Photoluminescence Quantum Yield Spectrometer C13534-11 from Hamamatsu. To be able to measure the PL QY, the films were deposited on quartz substrates. The films were excited by monochromatic light with a wavelength of 365 nm, and the emitted photons from the sample were collected by a linear image sensor with a range between 350 to 1100 nm. Kinetic curves of PL decay were registered using a custom-designed setup based on a FluoTime300 luminescence spectrometer (Picoquant) equipped with a 402 nm LDH-P-C-405B laser. The samples were excited by a 402 nm laser forwarded to the samples by an optical fiber, and the PL signal was collected in the range of 420–800 nm with excitation and emission slits set to 4 nm. Raman spectra were detected on a WITec alpha700 confocal Raman microscope equipped with a UHTS 300 spectrometer in a spectral range of 100–1100 cm^–1^ and a resolution of 2 cm^–1^. The samples were excited by a 532 nm laser with a maximal power of 7 mW.

## Abbreviations

CANBIC: Cs_2_Ag_*x*_Na_1–*x*_Bi_*y*_In_1–*y*_Cl_6_
CL: cathodoluminescence
LO: longitudinal optical
NCs: nanocrystals
PL: photoluminescence
QY: quantum yield
SSVD: single-source vacuum deposition
STE: self-trapped exciton
